# Impacto directo e indirecto del COVID-19 en la esperanza de vida al nacer de Chile en el año 2020

**DOI:** 10.1590/0102-311XES182823

**Published:** 2024-05-20

**Authors:** Gonzalo Ghío-Suárez, Andrés Alegría-Silva, Jenny García-Arias

**Affiliations:** 1 Instituto Nacional de Estadísticas de Chile, Santiago, Chile.; 2 Institut National d’Éétudes Démographiques, Paris, France.

**Keywords:** COVID-19, Respiratory Tract Diseases, Life Tables, Age-Specific Death Rate, Causes of Death, COVID-19, Enfermedades Respiratorias, Tablas de Vida, Mortalidad por Edad, Causas de Muerte, COVID-19, Doenças Respiratórias, Tábuas de Vida, Mortalidade por Idade, Causas de Morte

## Abstract

El artículo muestra el impacto directo e indirecto del COVID-19 en la esperanza de vida de Chile durante el año 2020, utilizando las estadísticas de defunciones definitivas publicadas en marzo del año 2023. Para ello, se estimó una mortalidad contrafactual para año 2020 sin el COVID-19, siguiendo el patrón de mortalidad según causas de muerte desde 1997 a 2019, se elaboraron tablas de mortalidad para calcular la esperanza de vida para los años 2015 a 2020 y para el año 2020 estimado, y luego se descompuso la diferencia entre la esperanza de vida esperada y observada del año 2020 según grupos de edad y causas de muerte. La esperanza de vida del año 2020 quiebra la tendencia a su aumento entre 2015 y 2019, mostrando un retroceso, en hombres y en mujeres, con respecto al año 2019, de 1,32 y 0,75 años respectivamente. Con respecto al año 2020 estimado, la esperanza de vida del 2020 observado es 1,51 años menor en hombres y 0,92 en mujeres, pero el impacto directo del COVID-19 en pérdida de esperanza de vida fue mayor, 1,89 para los hombres y 1,5 para las mujeres, concentrándose en las edades entre los 60 y 84 años en hombres y entre 60 y 89 años en mujeres. El impacto directo negativo del COVID-19 a la esperanza de vida en parte fue contrarrestado por impactos indirectos positivos significativos en dos grupos de causas de muerte, las enfermedades del sistema respiratorio y las enfermedades infecciosas y parasitarias. El estudio muestra la necesidad de distinguir los impactos directos e indirectos del COVID-19, por la incidencia que pueden tener en la salud pública cuando el COVID-19 baje su intensidad y se eliminen las restricciones de movilidad.

## Abstract (en)

This article shows the direct and indirect impacts of COVID-19 on life expectancy in Chile in 2020, based on mortality statistics published in March 2023. To this end, a counterfactual mortality was estimated for 2020 without COVID-19; based on the pattern of mortality by cause of death from 1997 to 2019, mortality charts were created to calculate life expectancy from 2015 to 2020 and an estimation for 2020, and the difference between expected and observed life expectancy in 2020 was then separated by age group and cause of death. Life expectancy in 2020 interrupted the upward trend from 2015 to 2019, showing a decline of 1.32 years in men and 0.75 years in women compared to 2019. Compared to the estimated 2020, life expectancy was 1.51 years lower in men and 0.92 years lower in women, but the direct impact of COVID-19 on the decrease in life expectancy was greater (1.89 for men and 1.5 for women) in the 60-84 age group in men and the 60-89 age group in women. The direct negative impact of COVID-19 on life expectancy was partially mitigated by significant positive indirect impacts on two groups of causes of death: diseases of the respiratory system and infectious and parasitic diseases. This study shows the need to differentiate direct and indirect impacts of COVID-19, due to the implications for public health when the intensity of COVID-19 decreases and mobility restrictions are suspended.

## Resumo

Este artigo apresenta os impactos direto e indireto da COVID-19 na expectativa de vida no Chile em 2020 a partir de estatísticas de mortalidade publicadas em março de 2023. Para tanto, foi estimada uma mortalidade contrafactual para 2020 sem a COVID-19; a partir do padrão de mortalidade por causa de morte de 1997 a 2019, foram criadas tabelas de mortalidade para calcular a expectativa de vida para o período de 2015 a 2020 e para o ano estimado de 2020 e, em seguida, a diferença entre a expectativa de vida esperada e observada em 2020 foi separada por faixa etária e causa de morte. A expectativa de vida em 2020 interrompe a tendência de aumento entre 2015 e 2019, mostrando um declínio com relação a 2019 de 1,32 ano nos homens e 0,75 ano nas mulheres. Com relação ao ano estimado de 2020, a expectativa de vida observada é 1,51 ano menor nos homens e 0,92 nas mulheres, mas o impacto direto da COVID-19 na diminuição da expectativa de vida foi maior (1,89 para homens e 1,5 para mulheres), concentrando-se nas idades entre 60 e 84 anos nos homens e entre 60 e 89 anos nas mulheres. O impacto direto negativo da COVID-19 na expectativa de vida foi parcialmente atenuado por impactos indiretos positivos significativos em dois grupos de causas de morte: doenças do sistema respiratório e doenças infecciosas e parasitárias. Este estudo mostra a necessidade de diferenciar impactos diretos e indiretos da COVID-19, devido às implicações para a saúde pública quando a intensidade da COVID-19 diminuir e as restrições de mobilidade forem suspensas.

## Introducción

En consonancia con la evolución mundial de la esperanza de vida al nacer (en adelante e(0)), en Chile, desde inicios del siglo XX y hasta el año 2019, la e(0) presentó un continuo ascenso. La disminución de la letalidad de las enfermedades infecciosas, digestivas y respiratorias en la población menor de cinco años de edad ^1^^,^^2^^,^^3^ es asociado al aumento de la e(0) en el país. Para el período entre el final del siglo XX y las dos primeras décadas del siglo XXI, esta tendencia continuó de manera sostenida, aunque a un ritmo más lento. Así, la e(0) estimada por el Instituto Nacional de Estadísticas de Chile (INE) para 2002 fue de 73,9 para los hombres y 80,0 para mujeres, mientras que en el año 2016 de 77,22 y 82,85 respectivamente ^4^.

En este escenario de disminución constante de la mortalidad, en marzo de 2020 llegó el primer caso de COVID-19 a Chile. Para final de año, el total de defunciones por COVID-19 fue de 18.680, lo que representa un 14,81% del total de 126.169 muertes ocurridas en Chile en ese año, y un aumento muy superior a los incrementos interanuales observados en los años anteriores, 15,06% respecto a las defunciones de 2019 ^5^. En respuesta a la emergencia nacional e internacional, se suspendieron las actividades educativas presenciales, y se decretaron cuarentenas en diferentes zonas del país durante distintos periodos del año. Las restricciones de movilidad duraron varios meses y, en algunos momentos, abarcaron a prácticamente todo el territorio nacional. Tanto la pandemia como las medidas en marcha para su mitigación cambiaron los patrones de mortalidad del país, dando como resultado la disminución de la esperanza de vida al nacer respecto a lo observado en años anteriores ^6^.

Evaluaciones previas del impacto del COVID-19 en Chile se han concentrado en estimar el exceso de muertes ocurrido durante la pandemia ^7^, los cambios observables en la esperanza de vida en comparaciones internacionales ^6^^,^^8^, así como también en calcular el diferencial de mortalidad entre los espacios urbanos ^9^ y el estatus socioeconómico de la población ^10^ para el año 2020. Sin embargo, estimaciones detalladas de los cambios en la distribución de las causas de muerte durante la pandemia del COVID-19 siguen estando pendientes en la agenda de investigación. En este sentido, el objetivo de este artículo consiste en medir el impacto directo e indirecto de la pandemia del COVID-19 en la esperanza de vida al nacer de Chile en el año 2020. Para ello, se hace uso de un análisis contrafactual que comparará la mortalidad observada del año 2020, con una mortalidad “esperada” calculada en base a la trayectoria de la mortalidad de las últimas décadas. La esperanza de vida al nacer es el indicador del nivel de mortalidad que se comparará en ambos escenarios. En primer lugar, se estima la e(0) desde el año 2015 al 2020, con la intención de cuantificar (en años) el quiebre ocurrido durante la pandemia del COVID-19 en la tendencia que llevaba la e(0). Luego se descompone la diferencia encontrada en ambas esperanzas de vida por grupos quinquenales de edad y causas de muerte, de manera de dar cuenta del impacto directo e indirecto del COVID-19 en la mortalidad del año 2020.

El impacto directo es medido a través de la incidencia en la e(0) de las defunciones cuya causa de muerte fue declarada como COVID-19, mientras que el impacto indirecto se presenta en variaciones significativas de las demás causas de muerte entre 2019 y 2020, variaciones que pueden ser el resultado tanto del rol del virus como acelerador de complicaciones ante cuadros mórbidos específicos, como de la reducción de la prestación de servicios de salud a pacientes con otras enfermedades durante la emergencia. Así también, da cuenta del impacto de medidas destinadas a la contención de la pandemia (como confinamientos, restricciones de circulación) que podrían afectar los patrones de ocurrencia de otras causas de muerte.

Nuestras hipótesis apuntan a encontrar una disminución significativa de la e(0), con una diferencia mayor entre la e(0) esperada y observada para los hombres que respecto a las mujeres, dado que la evidencia internacional señala un mayor impacto directo del COVID-19 para esta subpoblación ^11^^,^^12^^,^^13^. Además, esperamos encontrar impactos indirectos del COVID-19 en la mortalidad, causando variaciones significativas en otras causas de muerte, ya que han sido estudiados casos donde ciertas causas de muerte han disminuido producto de las restricciones de movilidad y otras medidas impuestas durante la pandemia ^14^.

## Métodos

De manera a observar la tendencia de la e(0) en Chile, se construyeron tablas de vida para el período de 2015 a 2020. Posteriormente, para realizar un análisis contrafactual, se contrastan dos escenarios para la mortalidad del año 2020; por una parte, un escenario con las defunciones observadas durante la pandemia, y por otra, un escenario con las defunciones esperadas, manteniendo los patrones de mortalidad chilenos observados en el período de 1997 a 2019; es decir, sin la ocurrencia de la pandemia de COVID-19. Finalmente, para observar el impacto específico del COVID-19 en la mortalidad, se analiza la diferencia entre la mortalidad esperada y observada en 2020, mediante la descomposición de la e(0) por edad y grupos de causas de muerte para cada sexo.

### Datos

Se usan las estadísticas de defunciones desde el año 1997 a 2020 publicadas por el Ministerio de Salud de Chile (MINSAL) y el INE ^15^ en marzo de 2023, en las cuales se establece un total de 18.680 muertes por COVID-19, cifra que viene a corregir a la baja las defunciones por esta enfermedad anteriormente informadas de manera provisional por el MINSAL, que ascendían a 22.218. En este sentido, se consideran las cifras oficiales más actualizadas de defunciones.

Por otra parte, los datos de nacimientos 2015 a 2020 provienen de los nacimientos corregidos que publica el INE, los cuales corresponden a los nacimientos ocurridos más el ajuste por la tendencia histórica de los “nacimientos tardíos”, es decir, aquellos registrados en años posteriores al de la ocurrencia (hasta el año X+7) ^16^.

Finalmente, la información de población para 2015 a 2020 se obtiene de las estimaciones y proyecciones de población base censo de 2017, elaborada por el INE ^17^. Todos los datos son públicos e informados por el INE.

### Mortalidad esperada

Para obtener la mortalidad esperada del año 2020, por grupos de causas de muerte, según grupos de edad y sexo, se siguió la siguiente secuencia: primero, se proyectó el número de muertes mensual por grupos de causas y sexo, a partir de la serie de defunciones mensuales entre los años 1997 y 2019. Dicha proyección se realizó por medio de series de tiempo mediante la función “auto ARIMA”, procedente del software R-Studio (http://www.r-project.org). Segundo, la proyección de la mortalidad total, por sexo, para el año 2020 se obtuvo a través de la suma de las proyecciones realizadas a nivel mensual por grupos de causas de muerte. Tercero, esta mortalidad anual por grupos de causa y sexo se distribuyó por edad según el patrón de mortalidad del año 2019. Mediante este procedimiento se obtuvo una mortalidad proyectada por causas de muerte para el año 2020 de 111.514 muertes esperadas, de las cuales, 58.412 son hombres y 53.102 mujeres. Los grupos de causas de muerte utilizados, los modelos y los errores asociados se pueden observar en la Tabla 1.

**Tabla 1:** Modelo ARIMA (modelo autorregresivo integrado de promedio móvil) y errores asociados, según la Clasificación Internacional de Enfermedades, versión 10 (CIE-10).

Grupo de causas	Hombres		Mujeres	
	Modelo serie de tiempo	Criterio de selección (Akaike)	Modelo serie de tiempo	Criterio de selección (Akaike)
Enfermedades infecciosas y parasitarias	ARIMA(0,1,1)(2,0,0)[12]	AIC = 2.197,70 AICc = 2.197,85 BIC = 2.212,17	ARIMA(1,1,2)(2,0,0)[12] con derivación	AIC = 2.079,38 AICc = 2.079,80 BIC = 2.104,70
Tumores malignos	ARIMA(0,1,1)(0,0,2)[12]	AIC = 2.898,79 AICc = 2.898,94 BIC = 2.913,26	ARIMA(1,1,1)(0,0,2)[12]	AIC = 2.836,77 AICc = 2.836,99 BIC = 2.854,85
Enfermedades endocrinas, nutricionales y metabólicas	ARIMA(2,1,2)(1,1,1)[12]	AIC = 2.380,36 AICc = 2.380,80 BIC = 2.405,36	ARIMA(0,1,1)(0,1,1)[12]	AIC = 2.378,54 AICc = 2.378,63 BIC = 2.389,25
Enfermedades del sistema circulatorio	ARIMA(2,0,2)(1,1,1)[12] con derivación	AIC = 2.881,57 AICc = 2.882,14 BIC = 2.910,18	ARIMA(1,0,1)(1,1,1)[12] con derivación	AIC = 2.925,05 AICc = 2.925,38 BIC = 2.946,51
Enfermedades del sistema respiratorio	ARIMA(1,1,2)(0,1,2)[12]	AIC = 2.865,53 AICc = 2.865,86 BIC = 2.886,97	ARIMA(1,0,2)(2,1,0)[12]	AIC = 2.921,67 AICc = 2.922,00 BIC = 2.943,13
Enfermedades del sistema digestivo	ARIMA(1,0,1)(2,1,1)[12] con derivación	AIC = 2.433,62 AICc = 2.434,06 BIC = 2.458,65	ARIMA(0,1,2)(0,0,2)[12] con derivación	AIC = 2.418,58 AICc = 2.418,89 BIC = 2.440,28
Enfermedades del sistema genitourinario	ARIMA(1,0,2)(0,1,1)[12] con derivación	AIC = 2.124,63 AICc = 2.124,96 BIC = 2.146,09	ARIMA(1,1,1)(0,0,1)[12]	AIC = 2.289,73 AICc = 2.289,88 BIC = 2.304,19
Otras causas	ARIMA(1,0,4)(0,1,1)[12] con derivación	AIC = 2.468,44 AICc = 2.469,00 BIC = 2.497,05	ARIMA(2,0,1)(0,1,1)[12] con derivación	AIC = 2.536,18 AICc = 2.536,51 BIC = 2.557,64
Afecciones originadas en período perinatal	ARIMA(0,1,1)	AIC = 1.894,44 AICc = 1.894,49 BIC = 1.901,68	ARIMA(2,1,1)(1,0,0)[12] con derivación	AIC = 1.760,32 AICc = 1.760,63 BIC = 1.782,02
Síntomas, signos y hallazgos anormales clínicos	ARIMA(3,0,0)(2,1,2)[12]	AIC = 2.220,00 AICc = 2.220,57 BIC = 2.248,61	ARIMA(3,1,3)(2,0,0)[12]	AIC = 2.404,48 AICc = 2.405,16 BIC = 2.437,03
Causas externas de mortalidad y morbilidad	ARIMA(1,1,1)(0,0,2)[12]	AIC = 2.778,93 AICc = 2.779,15 BIC = 2.797,01	ARIMA(0,1,1)(0,0,1)[12]	AIC = 2.467,16 AICc = 2.467,25 BIC = 2.478,01

AIC: criterio de información de Akaike; AICc: criterio de información de Akaike corregido; BIC: criterio de información bayesiano.

Fuente: elaboración propia, con base en los datos del Instituto Nacional de Estadísticas ^15^ (1997-2019).

### Tablas de vida

Las tablas de vida se construyeron en base a la mortalidad esperada y observada, por sexo y edad simple. Las tasas obtenidas fueron evaluadas y corregidas a través de la ecuación de equilibrio de Brass para las edades sobre los 6 años, y suavizadas mediante promedios móviles. Para la edad 0 se calcularon los factores de separación, en tanto, para la edad de 1 a 4 años se utilizaron los factores de Glover.

### Grupos de causas de muerte

Los grupos de causas de muerte considerados en el análisis se asocian a la definición etiológica de los capítulos de la Clasificación Internacional de Enfermedades, versión 10 (CIE-10). De esta manera, se toman 11 grupos de causas: COVID-19 (U00-U49), ciertas enfermedades infecciosas y parasitarias (A00-B99), tumores malignos (C00-D48), enfermedades endocrinas, nutricionales y metabólicas (E00-E90), enfermedades del sistema circulatorio (I00-I99), enfermedades del sistema respiratorio (J00-J99), enfermedades del sistema digestivo (K00-K93), enfermedades del sistema genitourinario (N00-099), causas externas de mortalidad y morbilidad (V01-Y98), síntomas, signos y hallazgos anormales clínicos (R00-R99) y otras causas. No se considera el grupo “P00-P96” (ciertas afecciones originadas en período perinatal) que, por su baja frecuencia, se suma a la agrupación “otras causas”.

### Tasas específicas observadas y esperadas por grupos de causas de muerte

**Figura 1:**
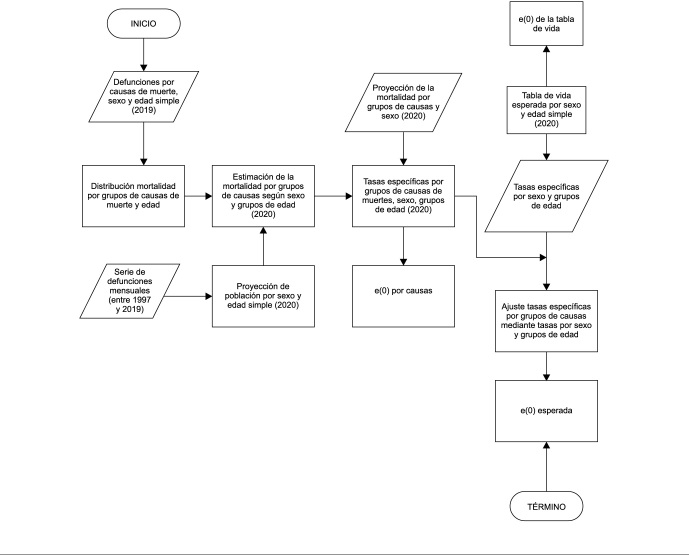
Tasas específicas de mortalidad esperada. Chile, 2020.

Las tasas específicas observadas consideradas en este estudio se obtienen de la mortalidad observada en el año 2020, según grupos de causas de muerte, sexo y grupos de edad. Por otro lado, las tasas específicas esperadas se conforman por la mortalidad esperada y las mismas estimaciones de población. La Figura 1 explica el flujo de procesos para la construcción de las tasas esperadas.

e(0): esperanza de vida al nacer.

Las tasas específicas por grupos de causas de muerte, según sexo y grupos de edad para ambos escenarios, se calibraron por las tasas específicas por edad anteriormente obtenidas de las tablas de vida observada y esperada, de manera tal que con las tasas específicas por grupos de causa se obtenga la e(0) definida por las tablas de vida.

### Descomposición de la e(0)

La descomposición de la e(0) se realiza utilizando el método *stepwise replacement*, propuesto por Andreev et al. ^18^. Este permite descomponer la diferencia entre la e(0) esperada y observada en términos de contribuciones (en años) de cada grupo de edad y grupo de causa de muerte. Este método mantiene el enfoque discreto de descomposición de Arriaga ^19^, en el que se promedian las diferencias encontradas entre dos esperanzas de vida para obtener un valor absoluto simétrico ^18^^,^^20^^,^^21^.

## Resultados

**Figura 2:**
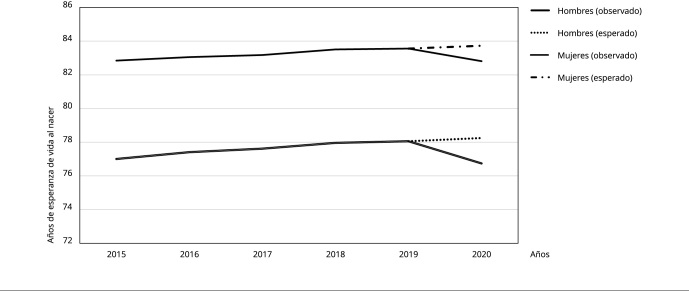
Evolución de la esperanza de vida al nacer en Chile entre 2015 y 2020.

La Figura 2 resume la evolución de las esperanzas de vida entre 2015 y 2020, en este se contrastan la e(0) observada entre los años 2015 y 2020, y la e(0) esperada de 2020, manteniendo los patrones de mortalidad del período entre 1997 y 2019; es decir, sin la ocurrencia de la pandemia de COVID-19. Entre 2015 y 2019, la e(0) observada de hombres y mujeres muestra una trayectoria estable, con incrementos interanuales continuos, aunque menores con respecto al año 2019. Así, entre 2015 y 2018 los incrementos en años de e(0) interanuales de hombres fueron de entre 0,21 y 0,41 y los de mujeres entre 0,13 y 0,33, para disminuir entre 2018 y 2019 a 0,1 y 0,05 respectivamente. El año 2020 esperado representa una continuidad en esta trayectoria, con alzas, respecto a 2019, de 0,19 años en hombres y 0,17 en mujeres.

Fuente: elaboración propia, con base en los datos del Instituto Nacional de Estadísticas ^15^^,^^17^.

El año 2020, observado claramente, se sale de esta trayectoria, mostrando una disminución brusca respecto a 2019, de -1,32 y -0,75 años en hombres y mujeres. Incluso, el año 2020 es menor, en ambos sexos, a 2015, en 0,26 y 0,03 años respectivamente, lo que marca un retroceso en la e(0) de, al menos, 5 años. Adicionalmente, el año 2020 también revierte la anterior tendencia a disminuir la brecha en la e(0) de hombres y mujeres. Esta brecha disminuyó por el aumento más acelerado de la e(0) de los hombres, lo que redujo la diferencia entre sexos de 5,84 años en 2015 a 5,5 en 2019. Por el contrario, en el año 2020 la diferencia aumenta a 6,07 años.

**Figura 3:**
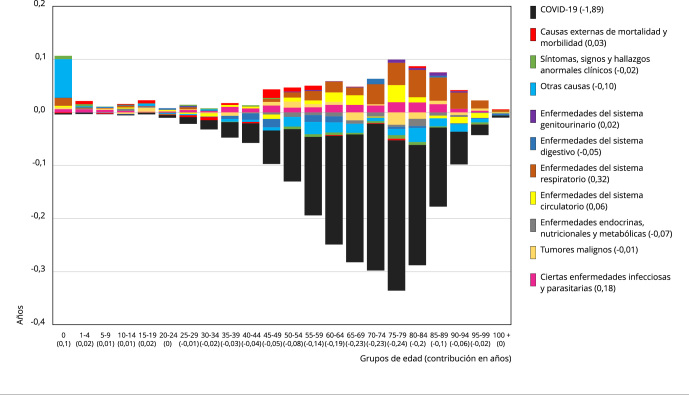
Contribución (en años) por causa de muerte y edad a la diferencia de la esperanza de vida al nacer esperada y observada de los hombres durante la pandemia del COVID-19. Chile, 2020.

Ahora bien, la diferencia en la e(0) observada y esperada en 2020 es descompuesta en años de contribución por cada grupo de edad y causa de muerte asociada, lo que se ilustra en las Figuras 3 y 4 para hombres y mujeres respectivamente. Las barras representan la contribución (en años) a la diferencia en e(0) de cada grupo quinquenal de edad. A su vez, las barras de cada grupo de edad se dividen según las contribuciones de las causas específicas de muerte a la diferencia entre ambas e(0). Como contribuciones positivas (por encima de cero) se entienden aquellas que disminuyen la diferencia entre la esperanza de vida al nacer observada y esperada; mientras que las contribuciones negativas (por debajo de cero) son aquellas que aumentan la diferencia.

Fuente: elaboración propia, con base en los datos del Instituto Nacional de Estadísticas ^15^^,^^17^.

**Figura 4:**
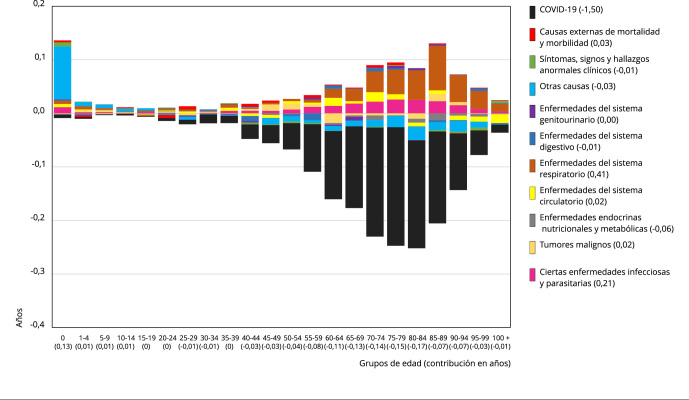
Contribución (en años) por causa de muerte y edad a la diferencia de la esperanza de vida al nacer esperada y observada de las mujeres durante la pandemia del COVID-19, año 2020.

Fuente: elaboración propia, con base en los datos del Instituto Nacional de Estadísticas ^15^^,^^17^.

El impacto directo del COVID-19 en los hombres, de -1,89 años, es más de un 20% superior al impacto en las mujeres, de -1,5 años. A la inversa, el resto de causas de muerte tienen una contribución positiva a la e(0) superior en las mujeres en casi un 40% por sobre los hombres, ya que en las mujeres suman +0,58 años y en los hombres +0,36. Estos dos factores sumados hacen que el impacto negativo de la mortalidad del año 2020 sobre la e(0) sea de un 40% superior en los hombres, en los cuales la e(0) tiene -1,5 años de diferencia entre el escenario esperado y el observado, en tanto en las mujeres es de -0,9 años.

La relación de ambos sexos con los grupos de causas de muerte es similar. Tanto hombres como mujeres tienen al COVID-19 como el principal vector de impacto negativo sobre la e(0). También, en ambos sexos, el grupo de enfermedades del sistema respiratorio y, en segundo lugar, el grupo de ciertas enfermedades infecciosas y parasitarias tienen las mayores contribuciones positivas en la e(0). En cuanto al conjunto de grupos de causas de muerte, en todos, excepto uno, el impacto sobre la e(0) es del mismo signo en ambos sexos, siendo la excepción el grupo de tumores malignos, que tiene un impacto negativo en hombres y positivo en mujeres.

En los grupos de edades, pese a que el gráfico de hombres (Figura 3) es más pronunciado en la parte del impacto negativo y el de mujeres (Figura 4) en el de las contribuciones positivas a la e(0), la forma que adquieren ambos gráficos es similar. En ambos se muestra una alta contribución positiva en el grupo de 0 años, y contribuciones positivas menores en los siguientes grupos de menor edad, hasta el grupo entre 15 y 19 años en hombres y el grupo entre 10 y 14 años en mujeres. En los siguientes grupos de edad los impactos negativos y las contribuciones positivas son muy bajos. Luego, a partir del grupo entre 35 y 39 años en el caso de los hombres y entre 40 y 44 años en el de las mujeres, comienzan a aumentar gradualmente los impactos en la e(0), especialmente los negativos, llegando a producirse los impactos más pronunciados, en hombres, en el grupo entre 75 y 79 años, y en mujeres, en los grupos entre 80 y 84 años para los impactos negativos y entre 85 y 89 años para las contribuciones positivas. Luego, en ambos gráficos los impactos disminuyen rápidamente en los grupos de más edad.

En cuanto al impacto del COVID-19 por grupos de edad, en hombres se observa a partir del grupo entre 25 y 29 años, y se incrementa con la edad hasta el grupo entre 75 y 79 años, para luego disminuir su impacto sobre la e(0). Las mayores contribuciones negativas del COVID-19 se observan entre los 60 y 84 años. En las mujeres, el COVID-19 también tiene un impacto negativo en la e(0) a partir del grupo entre 25 y 29 años, incrementándose progresivamente especialmente desde el grupo entre 40 y 44 años hasta el grupo entre 75 y 79 años, para luego disminuir dicho impacto a medida que se pasa a los grupos de edad mayores. En las mujeres, las edades con mayor impacto del COVID-19 en la e(0) es entre 60 y 89 años.

Finalmente, cabe revisar las hipótesis planteadas al inicio de la investigación en base a la información obtenida. La primera hipótesis apunta a la disminución significativa de la e(0) en el contexto de la pandemia del COVID-19, y a la mayor diferencia negativa entre la e(0) esperada y observada en los hombres que en las mujeres, lo cual ha sido ratificado, en el caso de Chile ambos sexos tuvieron una importante pérdida de años de e(0), siendo los hombres los que tuvieron una mayor pérdida; 1,5 años frente a 0,9 de las mujeres.

En relación a la segunda hipótesis, sobre el impacto indirecto del COVID-19 en los demás grupos de causas de muerte, dos grupos de causas de muerte producen impactos significativos, ambos positivos, en la variación de la e(0), las enfermedades infecciosas y parasitarias y las enfermedades del sistema respiratorio. Hay motivos para considerar que el COVID-19 tuvo un impacto indirecto en estos grupos de causas de muerte, por la importante variación que se produce entre el escenario esperado y el observado del año 2020. Los demás grupos muestran impactos positivos o negativos menores en la e(0) que pueden relacionarse con las fluctuaciones anuales habituales en el número de defunciones de cada causa, por lo que no estamos en condiciones de afirmar que sus variaciones se deban a efectos indirectos del COVID-19.

## Discusión

El estudio aquí presentado evalúa el impacto de la pandemia COVID-19 en la mortalidad en Chile durante el año 2020. Cuantificar el impacto de la pandemia COVID-19 requiere no solo el cálculo de la diferencia en la mortalidad del 2020 respecto al periodo anterior ^6^, sino también la consideración del incremento esperado en la e(0) para el año 2020 como parte de las pérdidas totales. Este escenario “esperado” sin pandemia COVID-19, en el que se proyecta continuidad de las tendencias históricas de la mortalidad, se debe a la necesidad de estudiar el impacto de la pandemia en el contexto de los incrementos interanuales en la e(0) como los que se han dado en Chile en los últimos años. La mera comparación de la mortalidad observada del año 2020 con periodos previos, por ejemplo, aquella de 2019 o el promedio del 2015 a 2019, subestimaría el impacto del COVID-19 sobre la tendencia histórica de la mortalidad. Razón por la cual se optó en esta investigación por un análisis contrafactual que permitiera contrastar lo observado en la pandemia versus lo potencialmente posible y esperado según los patrones de mortalidad. Esta evaluación más crítica daría cuenta de resultados que recrudecen el impacto de la pandemia en la esperanza de vida al nacer respecto a estudios que solo contabilicen el impacto medido en cambios respecto a periodos previos.

Así mismo, la construcción de un escenario “deseado” y no observable trae consideraciones metodológicas con efectos en las estimaciones realizadas. En este estudio se proyectó las tendencias históricas del número de defunciones por causas para estimar el escenario esperado de la esperanza de vida al nacer. Con esta estrategia, se mantiene la tendencia histórica de las causas de muerte; sin embargo, estas se estiman de forma independiente. Si bien es cierto que las muertes dependen directamente unas de otras a nivel agregado ^22^, se consideró dar mayor importancia a la tendencia inmediata en la distribución de las defunciones. Esta decisión se basa en la proximidad inminente del periodo proyectado que reduce cambios potencialmente abruptos en la tendencia de las defunciones por causas ^23^. Las decisiones tomadas en este sentido pueden ser discutidas y, eventualmente, mejoradas para desarrollar técnicas que puedan entregar mayor precisión en el análisis de impactos coyunturales en el número de defunciones en escenarios cambiantes de la mortalidad.

Una vez construidos dos escenarios comparables, se utilizó en este análisis el método *stepwise replacement* que permite descomponer (en años) las contribuciones por edad y causas específicas de muerte a la diferencia entre la esperanza de vida al nacer esperada y observada del 2020. La aplicación de este método de descomposición permite cuantificar el impacto directo derivado de las muertes por COVID-19 y el efecto que la pandemia tuvo en el riesgo de muerte por otras causas en consideración de su interdependencia. Se evitan así estimaciones derivadas de simulaciones por la eliminación o agregación de causas específicas en métodos de decrementos múltiples, típicamente utilizadas para calcular años de vida perdidos, y que afectan el principio de riesgo competitivo introducido por el COVID-19 sobre otras causas de muerte ^24^.

En este marco, es particularmente relevante el impacto positivo indirecto de la pandemia en el grupo de causas de muerte de enfermedades respiratorias y, en segundo término, en el de enfermedades infecciosas y parasitarias, los cuales tuvieron un fuerte descenso con respecto a 2019, tanto en hombres como en mujeres. Este descenso además representa un quiebre en la tendencia que presentaban ambos grupos en la mortalidad de Chile. El grupo de enfermedades respiratorias tenía una tendencia al alza desde hace más de 15 años, desde 2003 en hombres y 2002 en mujeres, años en los que alcanzaron los valores más bajos de la serie de 1997 a 2020 (3.728 y 3.668 muertes respectivamente), para luego subir consistentemente y alcanzar las 6.964 y 6.900 defunciones respectivamente. El grupo de enfermedades infecciosas y parasitarias alcanzó su valor más bajo en la serie, para hombres y mujeres, en el año 2008, llegando a 1.031 y 681 defunciones respectivamente, para luego también subir, en este caso con algunas fluctuaciones, hasta el año 2019, llegando a 1.402 y 1.161 defunciones. Así, el año 2020 presenta un descenso no esperable en base a la evolución que presentan ambos grupos de causas de muerte, ya que las enfermedades respiratorias generaron 5.202 muertes en hombres (una disminución en 25,3% con respecto a 2019) y 4.786 en mujeres (disminución de 30,64%), en tanto las infecciosas y parasitarias 1.231 decesos en hombres y 860 en mujeres (descensos de 12,2% y 25,93% respectivamente).

Ambos grupos de causas de muerte, donde se observan contribuciones positivas significativas en la e(0), son grupos en los que tiene alta incidencia el contagio entre personas en la propagación de las enfermedades. Así, medidas como las cuarentenas, las reducciones de la movilidad o la promoción del lavado de manos, implementadas para evitar el COVID-19, tuvieron un impacto en la incidencia de estos grupos de enfermedades, en su contribución a la mortalidad y en la distribución de las causas de muerte por edad y sexo. Sin embargo, en el caso de las enfermedades respiratorias, que muestran casi el doble de contribución positiva a la e(0) que las infecciosas y parasitarias, también puede haber incidido una mala clasificación de muertes por otras enfermedades respiratorias como muertes por COVID-19. Los posibles errores en la clasificación de las muertes por COVID-19 han sido señalados en otros estudios ^12^^,^^25^, que establecen un posible subregistro de las muertes por COVID-19 en México, además se han señalado los efectos que pueden tener en el número de muertes reportadas por COVID-19, por ejemplo, las diferentes definiciones de las muertes por COVID-19 utilizadas, las diferencias en las estrategias de testeos o en los sistemas de recolección de información. De esta manera, problemas en la clasificación de las causas de muerte deben ser considerados como una eventual explicación de las variaciones no esperadas en otras causas de muerte.

En los próximos años, estas diferentes posibilidades pueden tener incidencias muy distintas en la salud pública, particularmente en el contexto del levantamiento de las medidas de restricción a la movilidad de la población. Si el descenso de las muertes por estos grupos de enfermedades fue provocado por la baja de contagios, producto de la baja movilidad de la población, el descenso de muertes por estas causas puede revertirse en el contexto del levantamiento de restricciones, y la interacción de estas enfermedades con el COVID-19 puede provocar un alza de defunciones. Este escenario no sería así si el descenso se debiese a un mal registro de defunciones por causas respiratorias como provocadas por el COVID-19, en cuyo caso se habría exagerado el impacto de la pandemia por errores de registro. El análisis de las defunciones del año 2022, en el que ya no se aplicaron medidas de restricción de la movilidad, puede dar luz a esta incertidumbre.

## Conclusión

Para medir el impacto del COVID-19 sobre la esperanza de vida de Chile en el año 2020, se construyó, mediante series de tiempo, una estimación de la mortalidad de dicho año sin COVID-19. Este escenario esperado se contrastó con el observado, que mostraron las estadísticas definitivas de defunciones del año 2020 de Chile publicadas por el INE y el MINSAL en marzo de 2023 ^1^. Finalmente, se descompuso por sexo, edad y grupos de causas de muerte las diferencias encontradas en las esperanzas de vida al nacer de ambos escenarios. Este método permitió concluir que el COVID-19 tuvo un fuerte impacto directo que se muestra en altas tasas de mortalidad en la población mayor, particularmente de hombres, el cual fue contrarrestado parcialmente por un impacto indirecto que se reflejó en una disminución no esperable de las tasas de mortalidad en otros grupos de causas de muerte, particularmente las de los grupos de enfermedades infecciosas y parasitarias y del sistema respiratorio. Pese a este impacto indirecto positivo, el impacto general del COVID-19 se refleja en una fuerte disminución, en ambos sexos, de la esperanza de vida de Chile, que interrumpe décadas de continuos incrementos en este indicador del nivel de la mortalidad de la población.
